# Bi-allelic amplification of *ATM* gene in blastoid variant of mantle cell lymphoma: a novel mechanism of inactivation due to chromoanagenesis?

**DOI:** 10.1186/s13039-020-00526-x

**Published:** 2021-02-04

**Authors:** Veronica Ortega, Christina Mendiola, Juana Rodriguez, William Ehman, You-Wen Qian, Gopalrao Velagaleti

**Affiliations:** 1 Department of Pathology and Laboratory Medicine, UT Health San Antonio, San Antonio, TX USA; 2 Department of Pathology, University of Texas Medical Branch, Galveston, TX USA

**Keywords:** Mantle cell lymphoma (MCL), Blastoid mantle cell lymphoma (BMCL), Loss of heterozygosity (LOH), Single nucleotide polymorphism (SNP), Mutant allele specific imbalance (MASI), Copy number gain (CNG), Fluorescence in situ hybridization (FISH)

## Abstract

**Background:**

Mantle cell lymphoma (MCL) is derived from naïve CD5+ B-cells with the cytogenetic hallmark translocation 11;14. The presence of additional abnormalities is associated with blastoid variants in MCL (BMCL) and confers a poor prognosis. Many of these tumors also show deletion or loss of heterozygosity (LOH) of the *ATM* gene and biallelic *ATM* inactivation show significantly higher chromosomal imbalances.

**Case presentation:**

Here we report a 52 year-old male who presented to the clinic with worsening dyspnea, fever, chills, diffuse lymphadenopathy, splenomegaly and leukocytosis with blastoid cells circulating in blood. The bone marrow aspirate showed about 40% abnormal blast-looking cells and biopsy revealed a remarkable lymphoid infiltrate. The patient was diagnosed with blastoid variant mantle cell lymphoma (BMCL). Chromosome analysis on bone marrow showed a complex karyotype. FISH analysis from B-cell lymphoma panel showed bi-allelic amplification of *ATM* gene. Other abnormalities were present including *CCND1/IGH* fusion, confirming the MCL diagnosis, in addition to RB1 and p53 deletion. High resolution SNP-microarray studies showed complex copy number changes, especially on chromosomes 7 and 11, consistent with chromoanagenesis. Microarray studies also showed LOH at the *ATM* locus indicating the amplification seen on FISH is not biallelic.

**Conclusion:**

To the best of our knowledge, *ATM* gene amplification is not previously reported in BMCL and our case suggests a novel mechanism of *ATM* inactivation caused by chromoanagenesis resulting in mutant allele specific imbalance with copy number gain.

## Background

The hallmark of mantle cell lymphoma is the t(11;14) resulting in *CCND1/IGH* fusion leading to overexpression of *CCND1* [1]. Alterations of genes involved in cell cycle regulation, DNA damage response mechanisms and activation of cell survival pathways are closely linked to the aggressive and clinical behavior of MCL [2, 3]. The blastoid variant of MCL usually presents with more complex chromosome abnormalities in addition to the classic t(11;14) and patients with BMCL have poor prognosis [4]. The *ATM* (ataxia telangiectasia mutated) gene, also on chromosome 11q22q23 is known to play a significant role in MCL and frequent *ATM* gene inactivation has been confirmed in MCL [5]. A high proportion of MCL tumors show deletion or loss of heterozygosity (LOH) spanning the 11q22q23 region [5, 6]. Studies have shown that MCL tumors with inactivation of both *ATM* alleles (biallelic) have a significantly increased number of chromosome abnormalities compared to MCL tumors with wild type *ATM* alleles [5]. It is hypothesized that loss of *ATM* alleles increases chromosomal instability in these tumors [5]. The loss of ATM function may lead to tumorigenesis mostly through erroneous repair and generation of specific translocations in MCL [5]. In this context, we present an unusual case of *ATM* amplification which, based on FISH analysis, appears to be biallelic in nature. Since amplification usually results in over expression and not loss of expression, we undertook additional studies to explain this very unusual case of BMCL.

## Materials and methods

### Case presentation

A 52-year-old male with past medical history of pulmonary embolism, deep vein thrombosis (DVT) of left lower extremity, hepatitis C virus, intra venous drug abuse, presented to an outside hospital for worsening dyspnea, fever and chills, diffuse lymphadenopathy, splenomegaly and leukocytosis with blast appearing cells on smear review. Imaging studies with CT (computerize tomography) of thorax, abdomen, pelvis and scrotum showed no pulmonary embolism but DVTs were identified for which prophylactic Enoxaparin was given. He was noted to have bilateral pulmonary consolidations being worse on left, bilateral pleural effusions, massive splenomegaly and extensive retroperitoneal lymphadenopathies. Per CBC (complete blood count), there was markedly elevated WBC (white blood cell count), with blast appearing cells on smear review. Upon transfer to our clinic, patient was awake and alert, complained of dyspnea at rest, nonproductive cough, left upper quadrant abdominal pain, bilateral chest pain radiating to back, fever and chills.

CT scan demonstrated extensive retroperitoneal, pelvic and inguinal lymphadenopathies with massive splenomegaly. There was large area of decreased enchancement of the spleen, involving approximately 25% of the spleen, most likely splenic infarction. CBC at the admission showed a WBC 68.02 × 10^3^/ul, RBC 3.76 × 10^6^/ul, Hgb 10.2 g/dl, Hct 31.0%, MCV 82.4 fl, MCH 27.1 pg, Platelets 96 × 10^3^/ul. Differential count showed 25% Segs; 3% Bands; 8% Lymphocytes; 9% Monocytes; 1% Eosinophils; 0% Basophils; 2% Metamyelocytes; 1% Myelocytes; 0% Promyelocytes; 51% Blasts / blast appearing cells.

The blood smear showed leukocytosis due to the presence of blast appearing cells. These cells appeared medium to large in size with high N/C ratio, open nuclear chromatin pattern and distinctive nucleoli. Neutrophils were slightly left-shifted. Polymorphonuclear leukocytes (PMN) did not show dysplastic changes. Normocytic anemia and thrombocytopenia was also noted.

Bone marrow aspirate showed about 40% abnormal blast appearing cells with similar morphology as those in the blood. Bone marrow biopsy revealed remarkable lymphoid infiltrate in interstitial pattern and large lymphoid aggregates, contributing to about 40% of entire cellularity. The infiltrate was composed of mostly immature appearing cells with high N/C ratio and open chromatin. The abnormal cells were immunoreactive to CD20, PAX-5 and Cyclin D1, with a few scattered CD3 T-cells.

### Flow cytometry

Flow cytometry was performed on an 8-color BD FACSCanto II flow cytometer (BD Biosciences, San Jose, CA). Standard operating procedures (SOP) of flow cytometry immunophenotyping for lymphoma and leukemia was followed. Dotplot and histogram was created after data collection. CD45 vs side scatter was used to gate the lymphoid population. CD marker expressions of the abnormal lymphoid population was analyzed on multiple bivariant plots. Flow cytometry from the bone marrow aspirate detected a population (about 50% of total events) of lambda monoclonal B-cell population expressing CD19, CD22, and FMC-7 with coexpression of CD5, but negative for CD10, CD20, CD43, CD34 and CD38.

Based on these results, the patient was diagnosed with BMCL pending confirmation of chromosome and FISH analyses.

### Chromosome analysis

Cytogenetic analysis was carried out on bone marrow aspirate. Culture initiation, maintenance and harvest were performed using standard methods. Chromosomes were G-banded and then analyzed using a Cytovision image analysis system (Applied Imaging, Santa Clara, CA).

### Fluorescence in situ hybridization (FISH)

FISH was performed on the cultured biopsy specimen using a directly labeled dual color, dual fusion translocation probe *CCND1/IGH* (*IGH* labeled in spectrum green and *CCND1* in spectrum orange), *ATM* deletion probe (*ATM* labeled in spectrum orange and D11Z1 labeled in spectrum green), *RB1* deletion probe (*RB1* labeled in spectrum orange and CTB-163C9 probe in spectrum green), *IGH/BCL2* dual color, dual fusion probe (*IGH* labeled in spectrum green and *BCL2* in spectrum orange) and *TP53* deletion probe (*TP53* in spectrum orange and D17Z1 in spectrum green) (Cytocell, Windsor, CT). The probes were hybridized to interphase nuclei and metaphase chromosomes using standard procedures, followed by counterstaining with 4,6-diamidino-2-phenylindole, then analyzed using a Cytovision image analysis system (Applied Imaging, Santa Clara, CA). For interphase analysis, a minimum of 100 nuclei were scored, and for metaphase analysis, a minimum of 10 metaphases were scored.

### Single nucleotide polymorphism (SNP) oligonucleotide microarray

Given the complex nature of the abnormalities observed, chromosome microarray studies were carried out using Affymetrix CytoScan HD microarray. The Affymetrix CytoScan® HD Assay utilizes a high density combined CGH and SNP array platform, which assesses approximately 2,696,550 markers, including approximately 750,000 SNP markers. Each oligonucleotide is approximately 25 base pairs long. Intragenic probe spacing is approximately 1 probe every 880 base pairs and intergenic probe spacing is approximately 1 probe every 1700 base pairs. To perform the assay, gDNA is digested with the Nsp1 restriction enzyme and digested DNA is then ligated to Nsp1 adapters. The ligation product is then amplified via polymerase chain reaction (PCR) to produce amplicons in the 200–1100 bp range. The amplicons are then purified and digested with DNAse I to produce 25–125 bp fragments. The fragments are end-labeled with a modified biotinylated base and the sample is hybridized to the array. The array is washed and stained with a streptavidin-coupled dye and a biotinylated anti-streptavidin antibody. The array is scanned with the GeneChip Scanner and the signal intensity for each marker is assessed. Using the Chromosome Analysis Suite (ChAs 3.0) software, the signal for the sample is then compared to a reference set, which is based on the average of over 400 samples. Differences in signal between the sample and reference are expressed as a log2 ratio and represents relative intensity for each marker. A discrete copy number value is determined from the relative intensity data and is displayed. Genotype information for the SNP markers is visualized with the Allele Track [7].

## Results

**Fig. 1:**
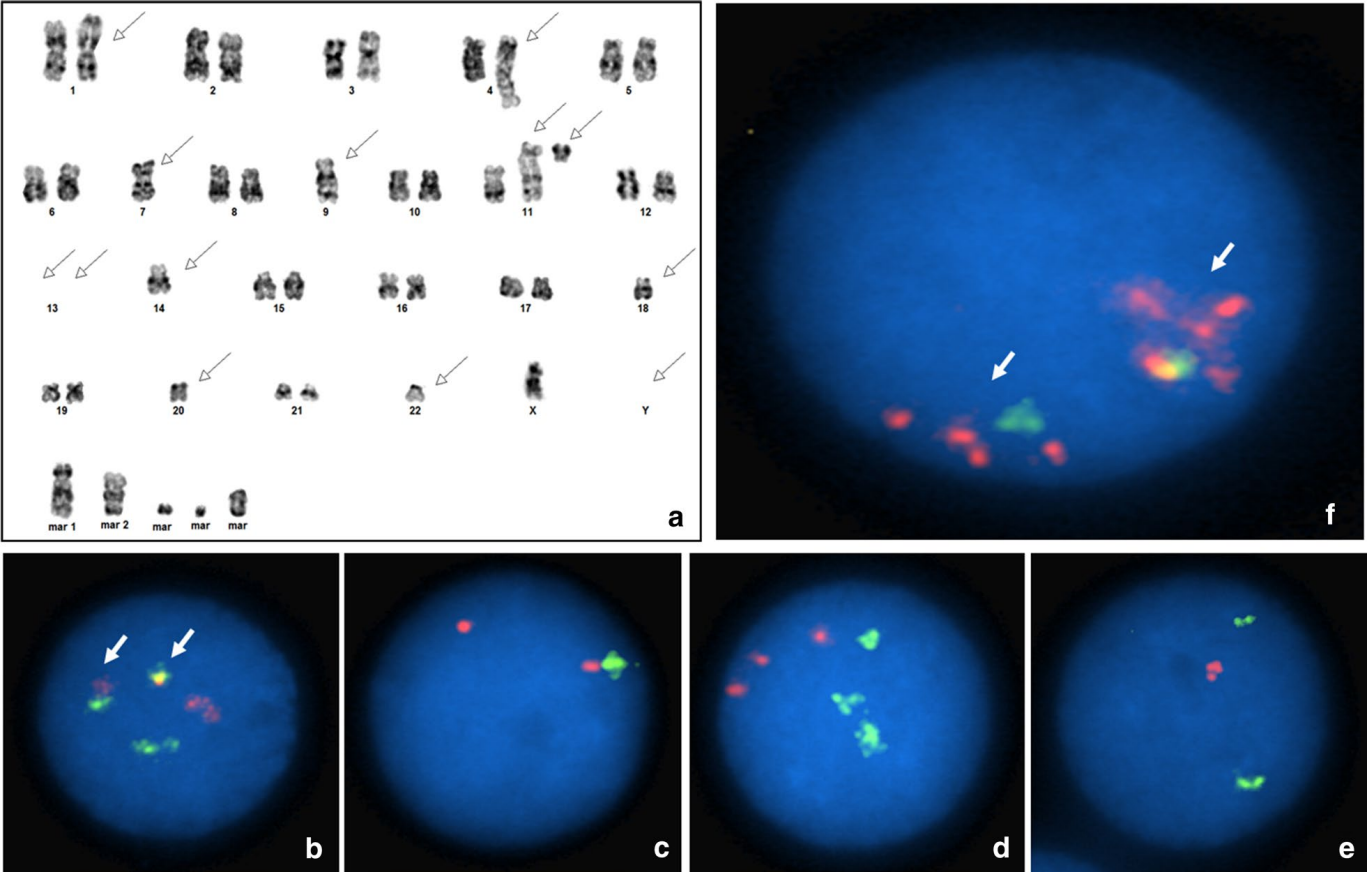
**a** G-banded karyotype showing complex abnormalities from the bone marrow. Arrows point to the abnormal chromosomes. **b** Interphase FISH showing the 2F/1O/1G pattern for the *CCND1* (orange) and *IGH* (green) probes indicating the CCND1/IGH fusion. Arrows point to the fusion signals. **c** Interphase FISH with *RB1* probe at 13q14 (orange) and CTB-163C9 at 13q34 (green) probes showing 2O/1G pattern indicating distal 13q deletion. **d** Interphase FISH with *IGH* (green) and *BCL2* (orange) probes showing 3O/3G pattern indicating gain of both *IGH* and *BCL2* gene regions. **e** Interphase FISH with *TP53* (orange) and D17Z1 (green) probes showing 1O/2G pattern indicating TP53 gene deletion. **f** Interphase FISH with *ATM* (orange) and D11Z1 (green) probe showing biallelic amplification of ATM gene region. Arrows point to the amplified *ATM* gene

Chromosome analysis on bone marrow showed a complex karyotype with multiple numerical and structural abnormalities including deletion of 1p, loss of 7 and 9, rearrangements of chromosome 11, loss of chromosome 13 and several markers. The karyotype was interpreted as 43 ~ 44,X,-Y,add(1)(p36.3),add(4)(q35),-7,-9,add(9)(q34), + 11,add(11)(q23),del(11)(q13),-13,-13,-14,-18,-22, + mar1, + mar2, + 1 ~ 4mar[cp6]/46,XY[14] [8] (Fig. 1a).

The results of interphase FISH analysis showed the characteristic *CCND1/IGH* fusion (Fig. 1b), distal 13q deletion (Fig. 1c), 3 copies of both *IGH* and *BCL2* gene regions (Fig. 1d) and *TP53* gene deletion (Fig. 1e). In addition, the *ATM* probe showed an unexpected pattern of biallelic amplification (Fig. 1f).

**Fig. 2:**
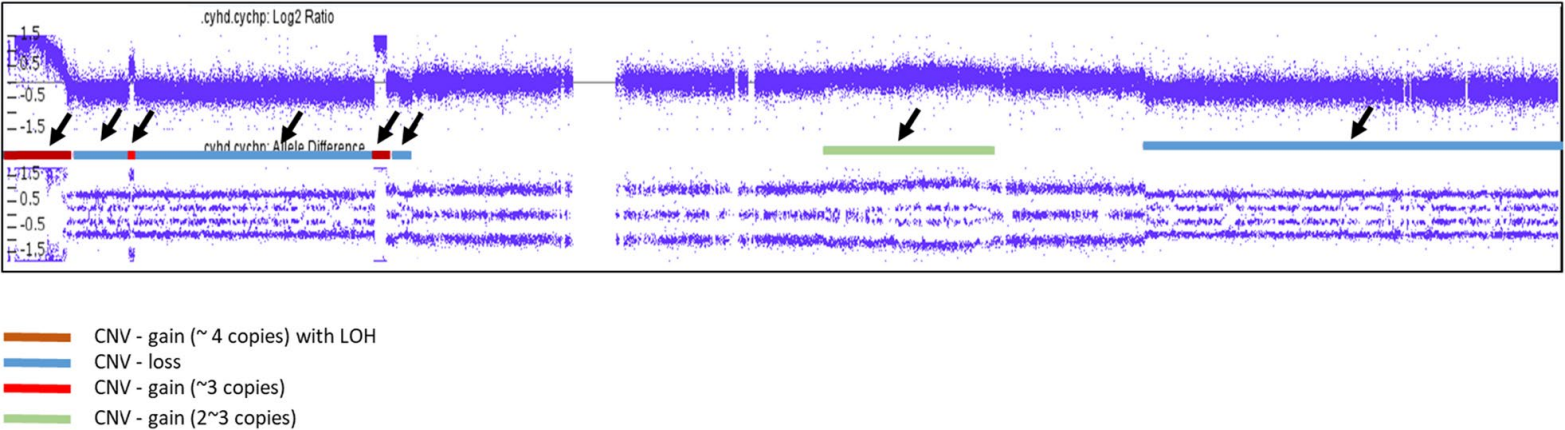
High-resolution SNP microarray showing complex CNV on chromosome 7. Arrows point to various CNV observed

**Fig. 3:**
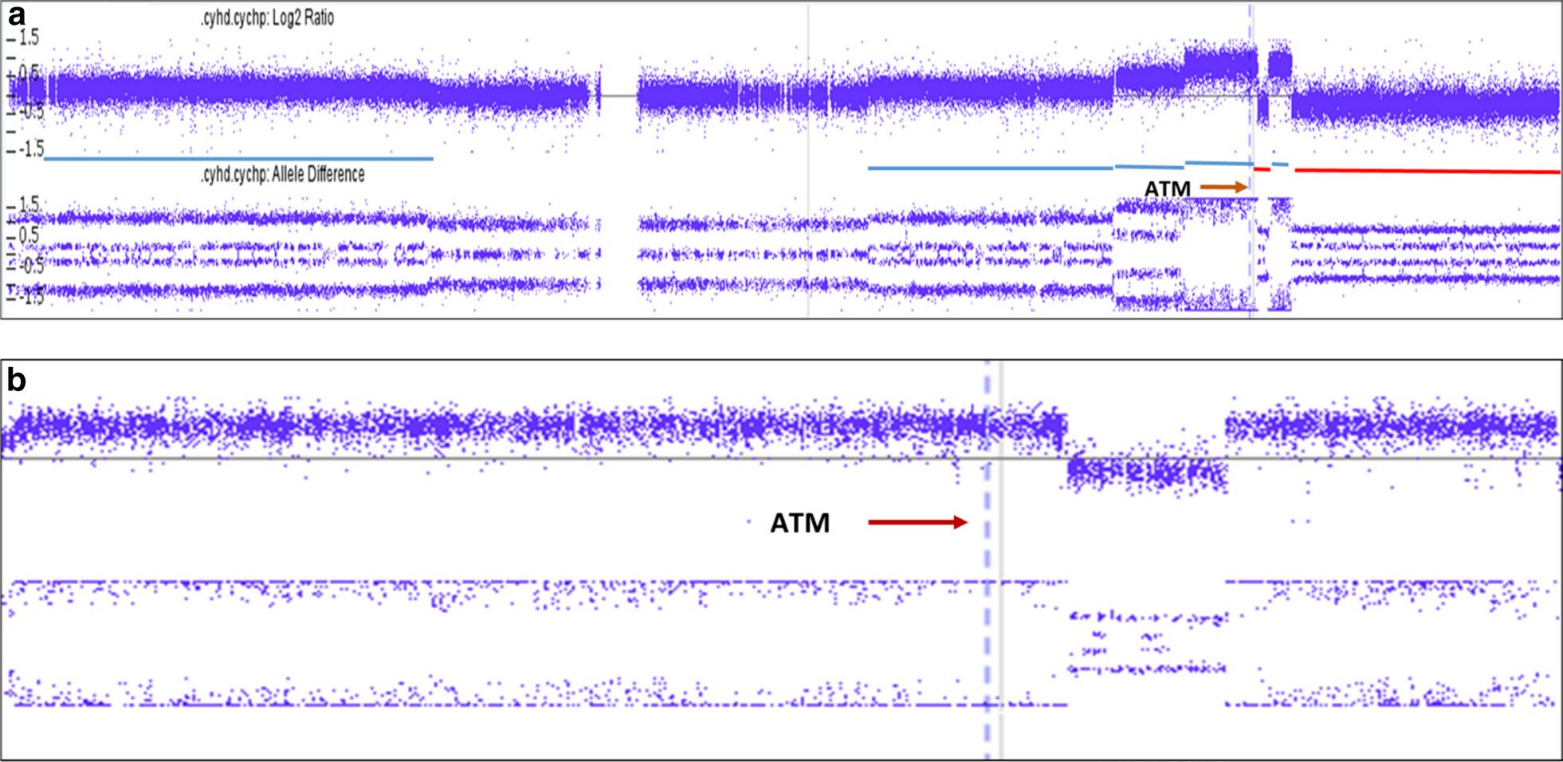
**a** High-resolution SNP microarray showing complex CNV on chromosome 11. Arrow points to the *ATM* locus. **b** Enlarged figure showing 11q22q23 region on high resolution SNP microarray showing LOH at the *ATM* locus. Arrow points to the *ATM* locus

Since the biallelic amplification of *ATM* gene has not been reported in BMCL, we performed additional high-resolution SNP-microarray (Affymetrix CytoScan) studies on the bone marrow aspirate to further characterize this finding. Microarray study demonstrated complex copy number variations consistent with abnormalities observed on routine chromosome analysis (Table 1). The pattern of complex multiple gains and losses, especially on chromosomes 7 (Fig. 2) and 11 (Table 1), is consistent with recently described phenomenon, chromoanagenesis [9]. Analysis of the chromosome 11q22q23 region, where the *ATM* gene is located, showed focal copy number gain consistent with the ATM amplification seen on FISH studies (Fig. 3a). However, SNP analysis of this region showed LOH for the entire region including the *ATM* locus (Fig. 3b).

**Table 1:** Results of high-resolution SNP Microarray

Chr	CNV	Cytoband start	Cytoband end	Genomic position start–end GRCh37	Size (Mb)	CN state
3	Gain	q13.11	q29	104,275,677–197,851,986	93.6	3
3	GainMosaic	q13.11	q29	104,347,395–197,851,986	93.5	3
4	LossMosaic	q13.1	q13.2	62,098,409–68,284,650	6.2	2
7	Gain	p22.3	p22.1	43,360–6,055,929	6.0	4
7	Gain	q21.13	q22.1	9,1027,069–100,918,485	9.9	3
7	Loss	p22.1	p21.3	7,263,512–12,522,467	5.3	1
7	Loss	p21.3	p14.1	13,107,394–37,647,617	24.5	1
7	Loss	q31.2	q36.3	116,762,426–159,119,707	42.4	1
7	GainMosaic	p14.2	p14.1	36,647,816–40,257,749	3.6	3
7	GainMosaic	q21.11	q22.1	85,370,550–103,219,749	17.9	3
7	LossMosaic	p21.3	p14.3	9,301,862–33,690,301	24.4	1
7	LossMosaic	q31.31	q36.3	117,402,248–159,119,707	41.7	1
9	Loss	p24.2	p13.2	2,582,103–38,044,560	35.5	1
9	LossMosaic	p24.2	p13.2	2,765,255–38,317,590	35.6	1
11	Gain	p15.5	p12	1,806,954–36,471,669	34.7	3
11	Gain	q13.4	q21	74,900,610–9,6287,065	21.4	3
11	Gain	q21	q22.2	96,439,368–102,413,943	6.0	3
11	Gain	q22.2	q22.3	102,414,120–108,729,175	6.3	4
11	Gain	q22.3	q23.1	109,718,291–111,689,133	2.0	4
11	Loss	q22.3	q22.3	108,729,392–109,715,602	9.9	1
11	Loss	q23.1	q25	111,731,307–134,788,683	23.1	1
11	GainMosaic	p15.5	p12	230,615–36,614,253	36.4	3
11	LossMosaic	q23.3	q25	114,847,579–134,938,470	20.1	1
13	Loss	q32.1	q34	95,258,943–114,609,241	19.4	1
13	LossMosaic	q12.3	q21.2	31,921,726–62,123,665	30.2	2
13	LossMosaic	q32.1	q34	96,138,381–115,107,733	19.0	1
14	Gain	q32.33	q32.33	106,072,250–106,692,891	0.6	3
17	Loss	p13.3	p11.2	525–21,565,553	21.6	1
17	LossMosaic	p13.3	p11.2	525–20,007,075	20.0	1
19	Gain	q13.12	q13.43	37,805,520–58,813,857	21.0	3
19	GainMosaic	q13.12	q13.43	37,516,085–58,956,888	21.4	3
X	Loss	p22.33	p21.3	2,693,466–25,047,578	22.4	1
X	Loss	p21.3	q23	25,048,192–115,589,080	90.5	1
X	Loss	q23	q24	115,592,089–118,552,562	3.0	1
X	Loss	q24	q28	118,552,991–155,059,506	36.5	1
Y	Gain	p11.31	q11.23	2,660,575–28,799,937	26.1	1

## Discussion and conclusions

To the best of our knowledge, we report the first case of what appeared to be a biallelic amplification of *ATM* gene in a patient with BMCL, which upon further studies with high-resolution SNP microarray is shown to be monoallelic with absence/loss of heterozygosity. Our case presents a novel mechanism of tumorigenesis in which the tumor cells acquire focal amplification of mutant *ATM* gene with loss of wild-type allele resulting in biallelic inactivation resulting in erroneous DNA repair and accumulation of complex chromosomal abnormalities and transformation to BMCL.

Recent studies brought to light two major mechanisms called chromothripsis [10] and chomoanasynthesis [11] to describe the occurrence of tens to hundreds of chromosomal rearrangements occurring within one or a handful of genomic regions. Recognizing that both these mechanisms produce complex, localized rearrangements, Holland and Cleveland [9] proposed the term chromoanagenesis to describe this class of chromosome rearrangements independent of the provoking mechanism. Chromoanagenesis is a catastrophic event resulting in complex chromosomal rearrangements at one or a few chromosomal loci [9]. Such focal inactivation of *RB1* gene due to chromothripsis has been reported in patients with retinoblastoma that lacked the point mutations or indels [12].

Unlike these published cases, the unique feature of our case is that while chromoanagenesis has caused multiple CNV on chromosome 11, the *ATM* gene locus is not involved in such complex rearrangements. On the other hand, the *ATM* locus was amplified in our patient, which is contrary to the published literature since amplifications are generally associated with over expression and not lack of expression. Since inactivation or loss of function of *ATM* locus is reportedly one of the major mechanisms for progression to MCL, we further investigated the apparent *ATM* amplification detected with FISH testing. The SNP patterns on the microarray suggested that while *ATM* is present in multiple copies, there is loss of heterozygosity (LOH) involving this locus. Studies have already showed that LOH involving the *ATM* gene region results in chromosomal instability and BMCL. As such, the unusual finding by FISH of biallelic amplification of *ATM* is in fact not biallelic, but is monoallelic with perhaps loss of wild-type allele.

Mutant allele specific imbalance (MASI) is originally coined to describe copy number alterations associated with mutant alleles of oncogenes. Recent advances in genomic analysis tools have further differentiated MASI into those with copy number gains (CNG), with copy neutral alterations (acquired uniparental disomy, UPD), or with LOH due to loss of the wild-type allele [13]. MASI is an established common occurrence in tumors and, in general, is considered an adverse prognostic indicator in tumors. Studies have shown that MASI with loss of wild-type allele can enhance or promote malignant growth.

**Fig. 4:**
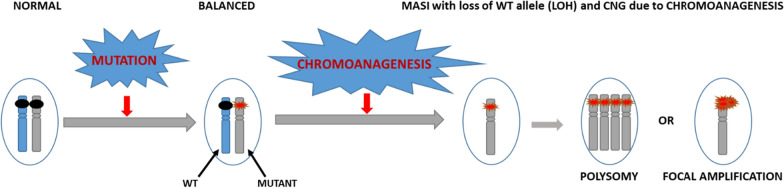
Schematic showing MASI in our case (Adopted from Yu et al. [13])

In light of these observations, we propose that a chromoanagenesis event on chromosome 11 caused MASI with loss of wild-type allele (Fig. 4) to explain the apparent amplification of *ATM* gene in our case with BMCL. The complex karyotype, especially with loss of 1p, 11q, 13q, 17p and X, that are often reported as most frequent additional abnormalities in MCL, further strengthens the hypothesis that *ATM* gene inactivation resulted in erroneous DNA replication and accumulation of complex chromosomal rearrangements.

One of the major limitations of our study is our inability to perform functional studies to determine the loss of expression of *ATM* gene. Non-availability of fresh patient sample for RNA extraction limited the options for additional functional studies. Another limitation of the study, in terms of limited availability of material in a clinical setting and lack of technical and other resources, does not allow us to investigate other possible hypotheses such as the complex rearrangements on chromosome 11 disrupting the *ATM* gene and the resulting haploinsufficiency contributing to loss of expression of *ATM* gene.

In summary, we report a unique case of BMCL with *ATM* amplification with LOH and propose that MASI with loss of wild-type allele due to chromoanagenesis resulted in loss of *ATM* expression leading to tumorigenesis.

## Data Availability

All relevant data and material is included in this publication.

## Abbreviations

MCL: Mantle cell lymphoma
BMCL: Blastoid variant of mantle cell lymphoma
FISH: Fluorescence in situ hybridization
MASI: Mutant allele specific imbalance
SNP: Single nucleotide polymorphisms
